# A case of gastric neuroendocrine tumor with an unusual morphology

**DOI:** 10.1007/s12328-026-02289-1

**Published:** 2026-02-16

**Authors:** Takuya Ohashi, Kenichiro Okimoto, Keisuke Matsusaka, Tomoaki Matsumura, Tsubasa Ishikawa, Hirotaka Oura, Tatsuya Kaneko, Yuki Ohta, Takasi Taida, Jun Kato

**Affiliations:** 1 Department of Gastroenterology, Graduate School of Medicine, Chiba University, Inohana 1-8-1, Chiba City, 260-8670 Japan; 2 Department of Diagnostic Pathology, Graduate School of Medicine, Chiba University, Chiba, Japan

**Keywords:** Neuroendocrine tumors, Autoimmune gastritis, Endoscopic submucosal dissection

## Abstract

Gastric neuroendocrine tumors (NETs), derived from enterochromaffin-like cells, commonly exhibit submucosal extension and manifest as submucosal tumor (SMT)-like lesions during endoscopy. We report a rare case of a gastric NET with endoscopical unusual pedunculated and reddish morphology. A 15-mm lesion in the gastric body showed histologically proved to be a grade G2 NET. Endoscopic submucosal dissection was performed, followed by surgery due to lymphatic invasion. Histological examination of the background gastric mucosa in the resected specimen revealed features of autoimmune gastritis, consistent with a type 1 gastric NET according to the Rindi classification. Recognition of such atypical morphology may aid diagnosis.

## Introduction

Gastric neuroendocrine tumors (NETs) originate from enterochromaffin-like (ECL) cells [1]. Although they are relatively rare, their incidence has been gradually increasing [1]. According to the 2019 World Health Organization (WHO) classification, NETs are categorized as grade G1, G2, or G3 based on the Ki-67 proliferation index, which reflects tumor prognosis [2]. Gastric NETs are typically classified into types 1–3 based on the scheme proposed by Rindi et al. in 1993, and treatment strategies are determined according to the disease type [3]. Gastric NETs typically originate from the deep mucosal layer and exhibit growth into the submucosa, often presenting as submucosal tumor (SMT)-like elevations [4]. Here, we report a rare case of a gastric NET exhibiting atypical pedunculated morphology.

## Case report

**Fig. 1:**
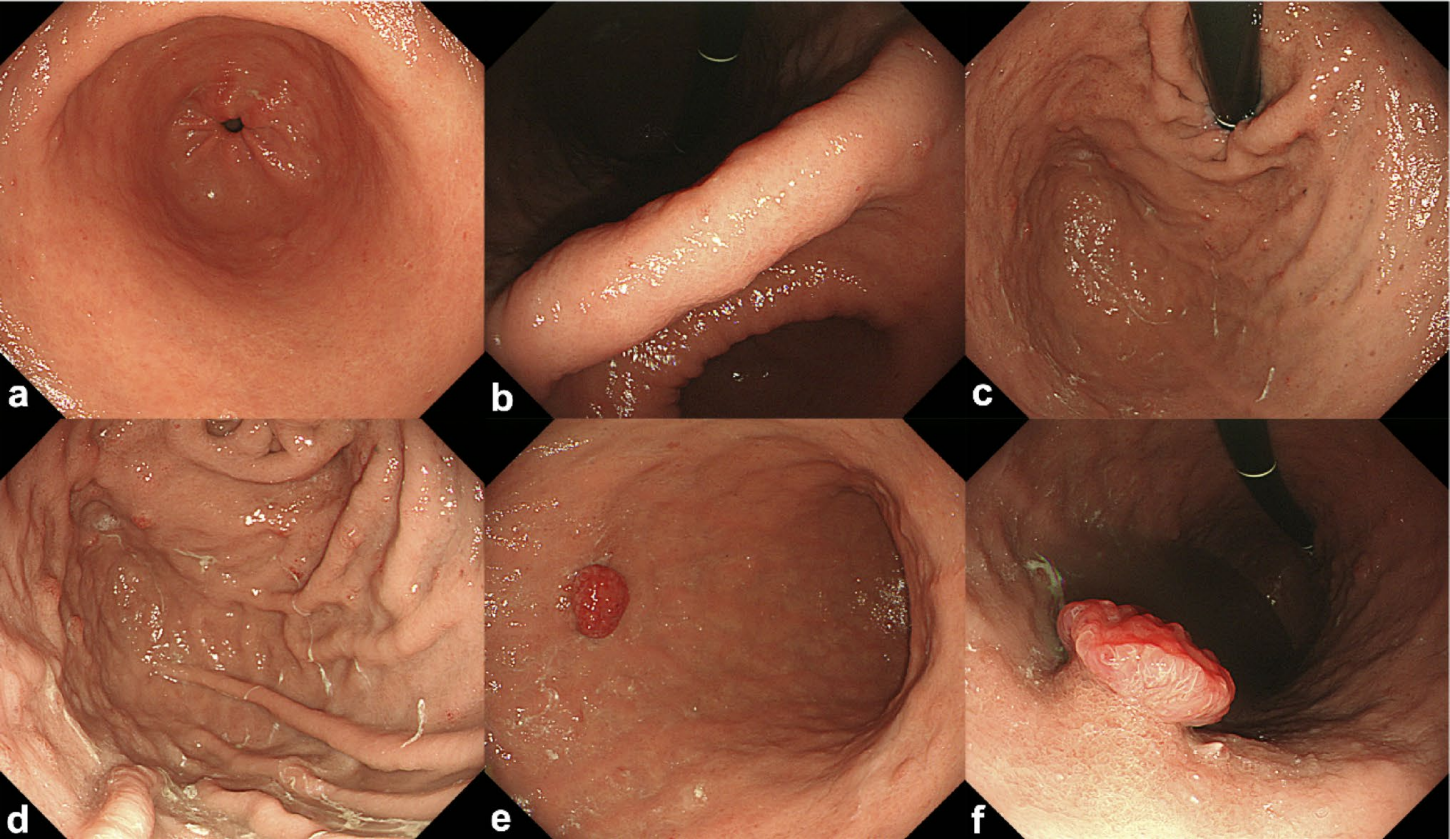
Conventional white-light endoscopic image of the entire stomach during upper gastrointestinal endoscopy. **a**–**e** Images of the antrum, angularis, fundus, and body of the stomach. No atrophic changes ware observed in the gastric antrum. On the other hand, atrophic changes were observerd throught the gastric body. An elevated lesion was detected along the greater curvature of the mid-gastric body. **f** The images of the lesion. The lesion appeared as a 15-mm pedunculated Ip + IIc-like lesion

**Fig. 2:**
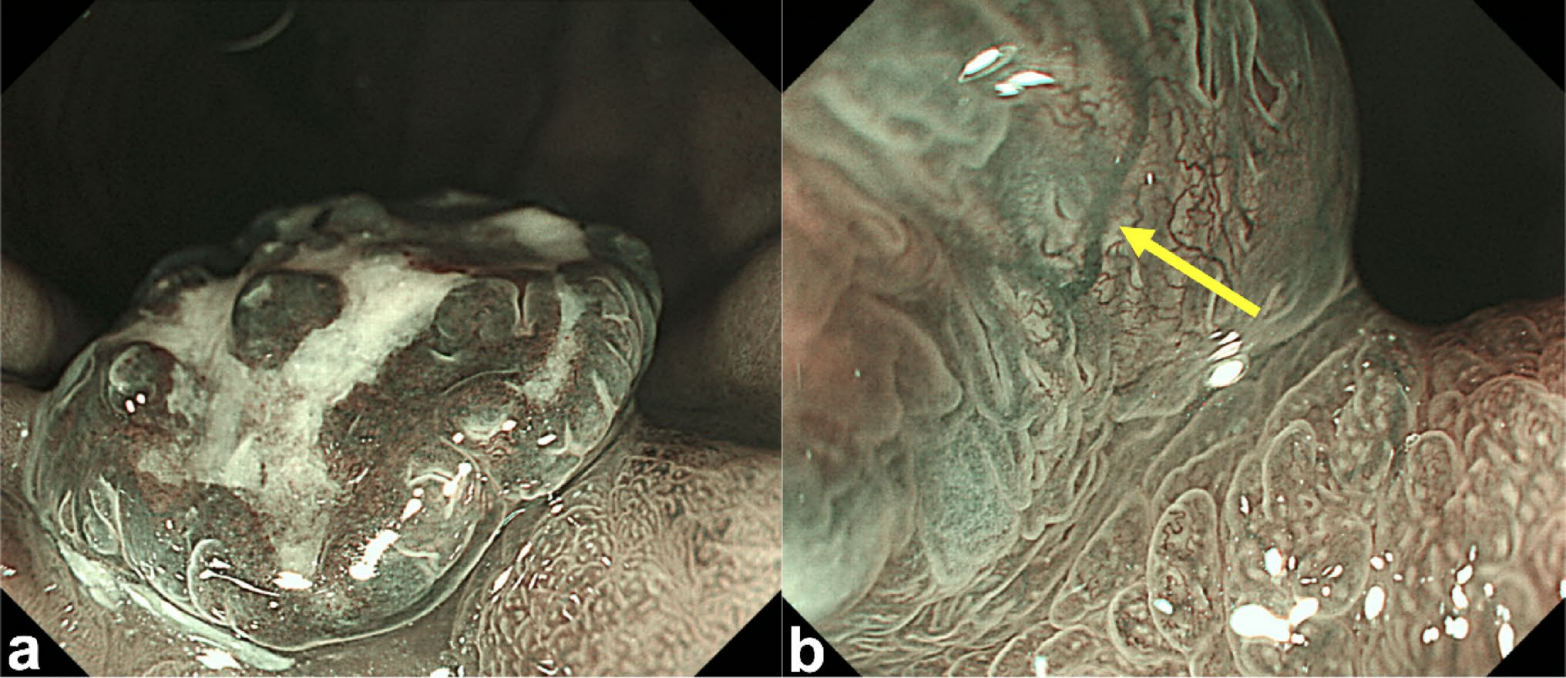
Narrow-band imaging (NBI) of the lesion. **a** Magnified image of the surface mucosa of the elevated lesion. There were few irregularities on the surface mucosa, and some areas were accompanied by slough and surface mucosal defects. **b** Magnified image of the edge of the elevated lesion. Although the lesion's margins were regular, vascular dilatation was observed. The yellow mark indicates vascular dilatation

The patient was a 48-year-old man with a history of simultaneous pancreas and kidney transplantation performed 12 years earlier for type 1 diabetes mellitus. This is a case following transplantation, and the patient had a history of oral administration of cyclosporine, prednisolone, and rabeprazole sodium. During a routine upper gastrointestinal endoscopy (UGE), a 15 mm pedunculated and reddish lesion was incidentally detected on the greater curvature of the mid-gastric body (Fig. 1). The patient had no history of *Helicobacter pylori* (*H. pylori*) eradication, and serum *H. pylori* antibody levels were < 3.0 U/ml (Table 1). In addition, the patient’s serum gastrin level was markedly elevated at 6300 pg/ml (Table 1). On conventional white-light endoscopy, the lesion appeared as a 15-mm pedunculated Ip + IIc-like lesion, with redness and slough areas on the surface mucosa. Narrow band imaging (NBI) with magnification revealed minimal surface irregularities, suggestive of hyperplastic or inflammatory changes (Fig. 2). Although the lesion's margins were regular, vascular dilatation was observed. Additionally, UGE revealed atrophic changes extending from the gastric body to the fundus (Fig. 1). On the other hand, no atrophic changes were observed in the antrum of the stomach (Fig. 1).

**Table 1:** Results of laboratory investigations on the first visit

Complete blood count		Biochemistry	
WBC	6900/μL	TP	6.8 g/dL
RBC	371 × 10^4^/μL	Alb	3.8 g/dL
Hb	10.7 g/dL	AST	25 IU/L
Hct	34.1%	ALT	18 IU/L
MCV	91.9 fL	LDH	218 IU/L
MCH	28.8 pg	ALP	361 IU/L
MCHC	31.4%	γGTP	10 IU/L
Plt	252 × 10^3^/μL	BUN	39 mg/dL
		Cre	2.12 mg/dL
Hormone		Na	138 mmol/dL
Gastrin	6300 PG/mL	K	4.2 mmol/L
		Cl	110 mmol/L
Immunity		T-Bill	0.5 mg/dL
*H. pylori* antibody	< 3 U/mL	CRP	0.09 mg/dL

The underlined values indicate abnormal findings

*Alb* albumin, *ALP* alkaline phosphatase, *ALT* alanine aminotransferase, *AST* aspartate aminotransferase, *BUN* blood urea nitrogen, *Cre* creatinine, *CRP* C-reactive protein, *FER* feritin, *γGTP* gamma-glutamyl transferase, *Hb* hemoglobin, *Hct* hematocrit, *LDH* lactate dehydrogenase, *MCH* mean corpuscular hemoglobin, *MCHC* mean cell hemoglobin concentration, *MCV* mean corpuscular volume, *Plt* platelets, *RBC* red blood cells, *T-Bil* total bilirubin, *TP* total protein, *WBC* white blood cell

**Fig. 3:**
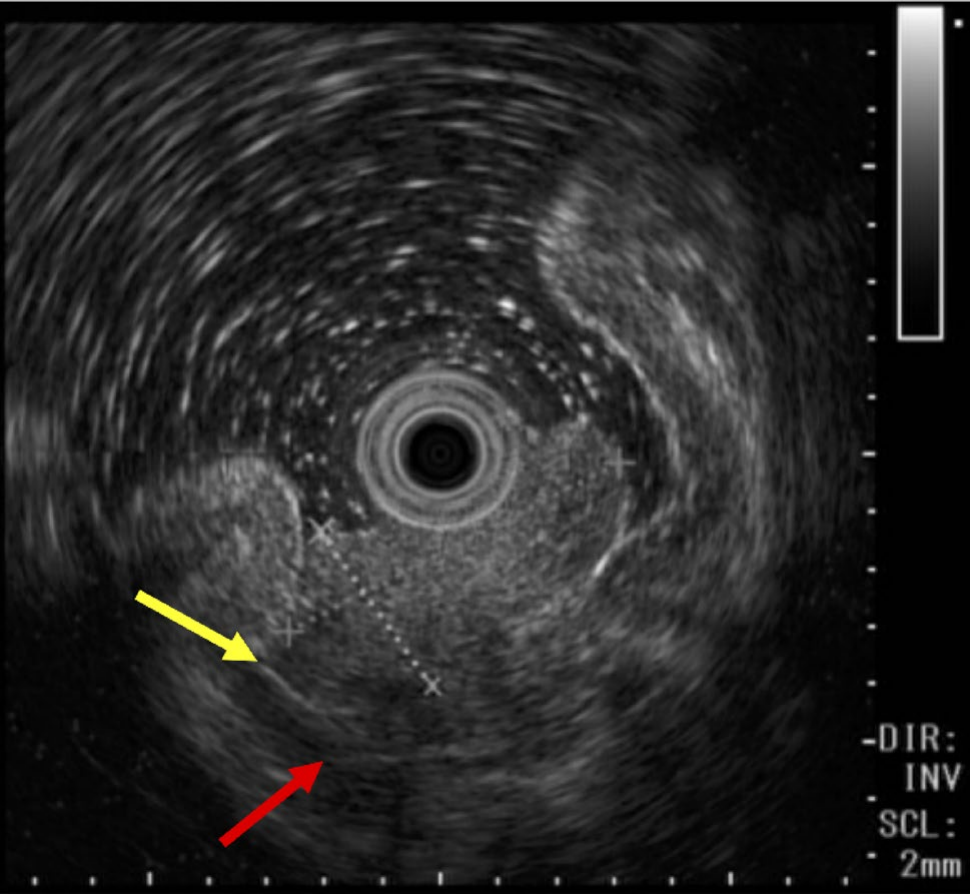
Endoscopic ultrasonography (EUS) images of the lesion. The hyperechoic area indicated by the yellow mark corresponds to the submucosal layer, while the hypoechoic area indicated by the red mark corresponds to the muscularis propria. EUS demonstrated a mixed-echo lesion originating from the proper mucosal or submucosal layer, raising concern for possible submucosal invasion. Although submucosal invasion by the tumor was suspected due to partial obscuration of the submucosal layer, the possibility of muscularis propria invasion was considered unlikely in this case, as there was no evidence of wall thickening in the muscular layer

Based on these endoscopic features, differential diagnoses included hyperplastic polyp and inflammatory polyp and tumor with malignant potential. However, histological examination of a biopsy specimen revealed a grade G2 gastric NET. Endoscopic ultrasound (EUS) demonstrated a mixed-echo lesion originating from the proper mucosal or submucosal layer, raising concern for possible submucosal invasion (Fig. 3). Given the patient's elevated perioperative risk, endoscopic submucosal dissection (ESD) was selected following informed consent. The lesion was completely resected under hospitalization. During endoscopic treatment, although partial fibrosis was observed in the submucosal layer, the lesion was successfully resected en bloc without technical difficulty.

**Fig. 4:**
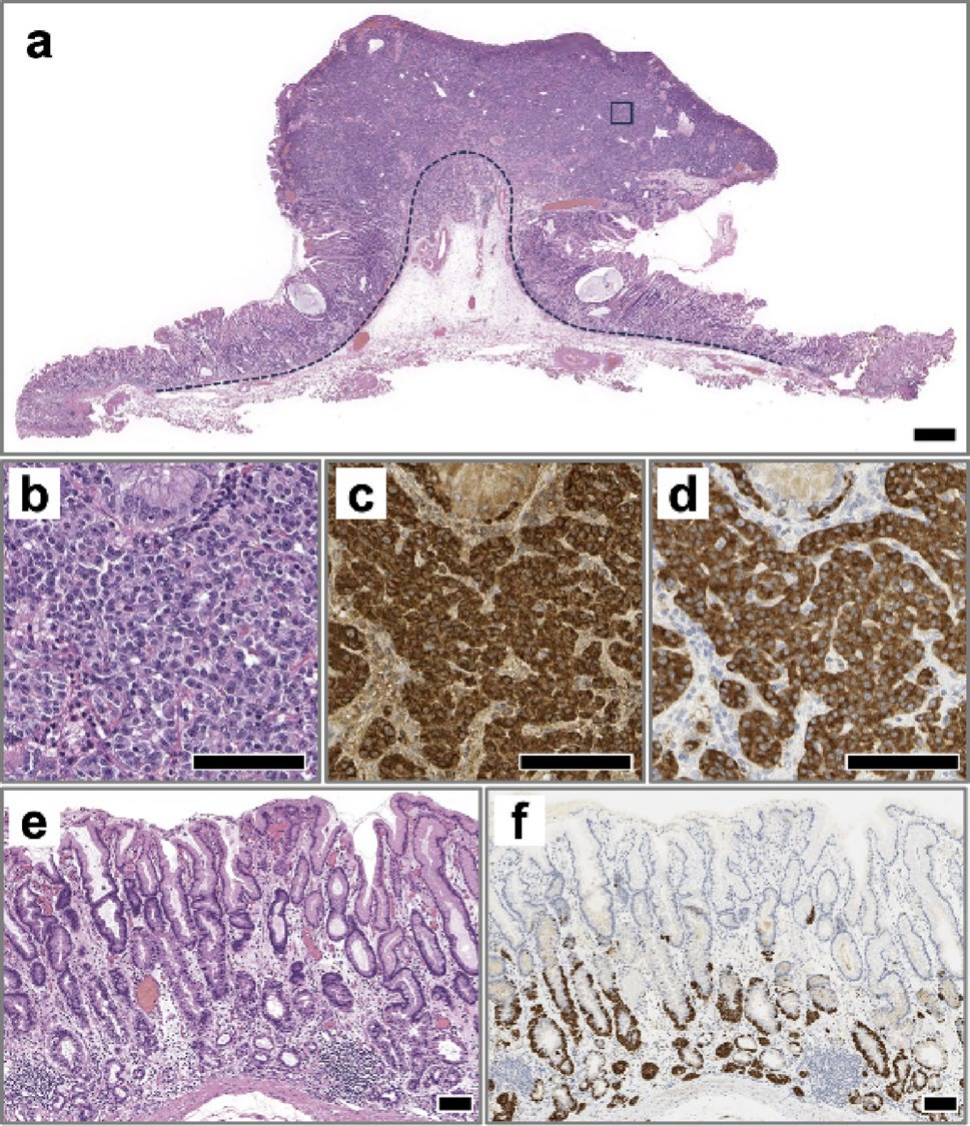
Pathological images of endoscopic submucosal dissection (ESD) specimens. **a** Low-power view of a gastric G2 NET stained with hematoxylin and eosin (H&E). The main lesion is located within the lamina propria and forms a subpedunculated polypoid mass. The black dotted line indicates the muscularis mucosae, and the black square corresponds to the area shown in Figures b–d. Scale bar: 1 mm. **b**–**d** High-power views of the boxed area in Figure a. **b** H&E staining reveals solid nests of atypical cells with round to oval nuclei, proliferating in a trabecular pattern with prominent intervening capillaries. **c**, **d** Immunohistochemistry for chromogranin A (**c**) and synaptophysin (**d**) shows strong cytoplasmic positivity, confirming neuroendocrine differentiation. Scale bars: 100 μm. **e**, **f** Background gastric mucosa. **e** H&E staining demonstrates severe atrophy of the fundic glands and foveolar hyperplasia in the superficial mucosa. ECMs and increased numbers of ECL cells are observed in the deep mucosa. **f** Immunohistochemistry highlights the distribution of ECL cells and ECMs. Scale bars: 100 μm

Histopathological analysis confirmed a grade G2 NET (Ki-67 labeling index 17.7%), tumor size 12 × 17 mm, staged as pT2 (Union for International Cancer Control 8th edition), Ly1 (D2-40), V0 (EVG), with negative horizontal (pHM0) and vertical (pVM0) margins (Fig. 4a–d). Examination of the background mucosa in the ESD specimen revealed marked atrophy of the fundic gland mucosa, with dense infiltration of lymphocytes and plasma cells in the deep lamina propria. There was also an increase in ECL cells and the presence of endocrine cell micronests (ECMs) (Fig. 4e, f). Due to the presence of lymphatic invasion, the patient subsequently underwent laparoscopic distal gastrectomy. No residual tumor or lymph node metastasis was identified in the surgical specimens.

## Discussion

We recently encountered a rare case of a gastric NET exhibiting pedunculated and reddish morphology. Endoscopic examination revealed advanced corpus dominant mucosal atrophy and histopathological analysis of the resected specimen showed ECMs in the deep mucosal layer. Although serum anti-parietal cell and anti-intrinsic factor antibodies were not measured, the combination of endoscopic and histological findings strongly suggested the presence of underlying autoimmune gastritis, meaning Rindi type 1 NET. In this case, the patient was taking proton pump inhibitor (PPI), but the serum gastrin level was 6,300 pg/ml, which is more than 60 times the normal value. Even considering the effect of PPI, this level was abnormally high, suggesting the possibility of autoimmune gastritis.

For Rindi type 1 gastric NETs larger than 10 mm, surgical resection is recommended in Japan [5]; however, in Europe, endoscopic treatment is considered an acceptable option in the absence of muscularis propria invasion [6]. In the present case, ESD was selected after careful consideration of the patient’s general condition. Regarding additional surgical treatment, the Japanese guidelines recommend distal gastrectomy or total gastrectomy with D2 lymph node dissection [5]. Previous reports have shown that antrectomy for Rindi type 1 gastric NETs can correct hypergastrinemia and lead to tumor regression [7]. In this case, taking into account the potential postoperative decline in quality of life in daily living, distal gastrectomy including the antrum was selected. Because there remains a risk of recurrence of NETs in the remnant stomach, regular postoperative endoscopic surveillance was considered necessary.

Gastric NETs, which arise from ECL cells, typically expand toward the submucosa and present as SMT-like protrusions under endoscopy [4]. These lesions often appear red or normal in color and may exhibit surface vascular dilation [4]. In this case, vascular dilation was observed, as in typical cases; however, the presence of a pedunculated and reddish morphology, complicating diagnosis based on the usual morphological features of gastric NETs. Endoscopic findings in this case revealed inflammatory changes on the surface of the elevated lesion, characterized by a mixture of redness and whitish coating, without any evidence of tumor exposure on the mucosal surface. In contrast, histopathological evaluation of the resected specimen showed denudation of the surface epithelium with tumor cells exposed at the surface. The denudation of the surface foveolar epithelium may have been caused either by active erosion present at the surface or by an artifact introduced during the resection process.

Although reports of pedunculated gastric NETs are extremely rare, a case report by Takeda et al. described an indistinct boundary between the head and stalk, with the head of the tumor appearing more reddish than the stalk [8]. In the present case, the boundary between the head and neck was also unclear, and similar redness of the head was observed; however, the degree of redness was more pronounced than that reported previously [8]. Histopathological findings of the head showed that the tumor extended and was distributed closer to the surface than in previously reported cases and was accompanied by abundant proliferation of dilated capillaries, which was considered to have resulted in the marked reddish mucosal appearance [8]. Furthermore, while vascular dilation was observed only in the head on NBI in the previously reported case, vascular dilation was observed only in the neck in the present case. Typical vascular dilation in gastric NETs is thought to result from expansive growth of the tumor in the submucosa, leading to stretching of the superficial capillaries [4]. Because no tumor component was present in the superficial layer of the neck in this lesion, it was presumed that the superficial capillaries were stretched in a manner similar to that seen in typical cases, resulting in the observed vascular dilation.

Another report of a pedunculated gastric NET suggested that, in cases where the tumor does not invade the muscularis propria, repeated peristaltic activity may cause gradual separation between the tumor and the underlying muscle layer, ultimately resulting in a pedunculated appearance [9]. In this case, the lesion was located on the greater curvature of the gastric body. Frequent mechanical stimulation due to peristalsis at this site may have contributed to the development of the pedunculated form. In the pathological findings of the resected specimen from this case, the tumor was primarily located within the mucosal layer, and its grossly pedunculated appearance was likely due to this localization. However, the mechanism by which the tumor developed toward the mucosal surface rather than the deeper layers remains unclear. Accumulation of additional similar cases may lead to new insights into this growth pattern in the future.

In conclusion, recognizing that gastric NETs can occasionally exhibit unusual pedunculated and reddish morphology may aid in accurate diagnosis.
